# Investigating the Dynamics of MCMV-Specific CD8^+^ T Cell Responses in Individual Hosts

**DOI:** 10.3389/fimmu.2019.01358

**Published:** 2019-06-19

**Authors:** Michael Gabel, Nicolas S. Baumann, Annette Oxenius, Frederik Graw

**Affiliations:** ^1^Center for Modelling and Simulation in the Biosciences, BioQuant-Center, Heidelberg University, Heidelberg, Germany; ^2^Institute of Microbiology, Department of Biology, ETH Zurich, Zurich, Switzerland

**Keywords:** CD8^+^ T cells, MCMV-infection, memory inflation, mathematical modeling, individual dynamics

## Abstract

Infection by Cytomegalovirus (CMV) is characterized by the massive expansion and continued maintenance of CMV-specific CD8^+^ T cells for certain CMV-derived peptides. This phenomenon called “memory inflation" has made CMV a primary target for the generation of T cell based vaccine vectors against various diseases. However, many aspects concerning the generation and maintenance of the inflationary CD8^+^ T cell response still remain to be resolved. In this study, we combined experimental data and mathematical models to analyze the dynamics of circulatory inflationary CD8^+^ T cells within individual mice infected by MCMV. Obtaining frequent measurements on the number and frequency of CMV-specific CD8^+^ T cells up to 70 days post infection, we find that mathematical models assuming differing viral stimuli during acute infection and the inflationary phase provide a better description for the observed dynamics than models relying on similar viral stimuli during both phases. In addition, our analysis allowed a detailed quantification of the different phases of memory inflation within individual mice (1^st^-expansion - contraction - 2^nd^ expansion/maintenance) indicating remarkable consistency of the timing of these phases across mice, but considerable variation in the size of the individual responses between mice. Our analysis provides a first step toward generating a mechanistic framework for analyzing the generation and maintenance of inflationary CD8^+^ T cells while accounting for individual heterogeneity. Extending these analyses by incorporating measurements from additional compartments and more prolonged sampling will help to obtain a systematic and quantitative understanding of the factors regulating the process of memory inflation.

## 1. Introduction

Cytomegaloviruses (CMVs) are a group of doubled-stranded DNA viruses that are known to cause life-long persistent infections in different mammalian species, including mice (MCMV), rhesus monkeys (RhCMV) and humans (HCMV) (1–3). Infection by this virus leads to a systemic infection in the host with the virus being able to spread and replicate within different target tissues, such as the spleen, lung and liver (4, 5). After an acute infection phase, CMV establishes a state of latency, preferentially hiding in the salivary glands and endothelial cells (6–8), with sporadic reactivations assumed to maintain a persistent infection and allowing for horizontal transmission to other susceptible hosts (9–11). Infection by CMV is usually well controlled by the host's immune system except for individuals with impaired cellular immunity who experience substantial pathology. An important hallmark of the immune response against CMV is the expansion and continued maintenance of large numbers of CMV-specific memory CD8^+^ T cells, a phenomenon called “memory inflation" (12–15). Due to these elevated, effector-memory type T cell levels, in combination with the ubiquity and general harmlessness of the virus, CMV is intensively investigated as a potential T-cell based vaccine vector for different diseases, e.g., including HIV and SIV infection (16–19). However, many questions regarding the generation, dynamics and maintenance of the inflationary CD8^+^ T cell response still need to be resolved.

**Figure 1:**
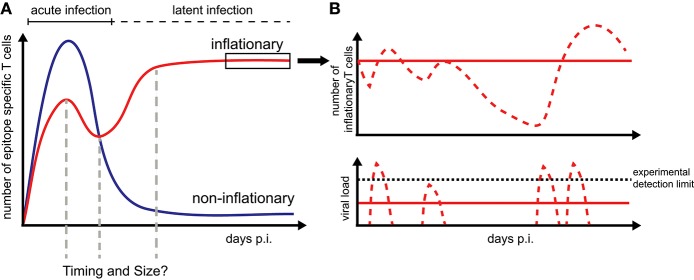
CD8^+^ T cell dynamics during CMV infection: **(A)** Infection by CMV induces inflationary (red) and non-inflationary CD8^+^ T cell responses (blue). Inflationary responses are dominated by a high number of effector and effector memory T cells during latent infection, with the factors regulating the timing and size of the responses within individual hosts still unresolved. Non-inflationary responses follow ‘classical’ memory dynamics with a decline in numbers and maintenance of mostly central memory T cells once the acute infection is resolved. **(B)** Inflationary responses can be maintained by different viral reactivation schemes during latency with continuous viral expression below the detection limit resulting in more stable inflationary responses (red solid lines) compared to oscillating or variable T cell dynamics given sporadic reactivation (red dotted lines).

Memory inflation only applies to certain CMV-derived peptides while other CMV-specific CD8^+^ T cells follow the conventional, i.e., non-inflationary dynamics, consisting of an initial expansion phase that is followed by a considerable contraction and long-term maintenance of central memory-type T cells (T_CM_) (Figure 1). Inflationary CD8^+^ T cells differ in phenotype and number from these “classical” memory T cells, maintaining a large number of effector-memory type T cells (T_EM_) over long periods of time. The half-life of these inflationary CD8^+^ T cells has been determined at 45–60 days within circulation (20) and up to 10–12 weeks within peripheral tissues (21). Thus, the constant replacement of lost cells is required to maintain the expanded T cell level (20, 21). Different, non-exclusive hypotheses exist to explain the generation and especially long-term maintenance of inflationary CD8^+^ T cells, mainly based on observations of MCMV infection in mice [reviewed in Klenerman and Oxenius (22)]. Torti et al. found that a proportion of T_CM_ cells in secondary lymphoid organs are dividing rapidly during latent MCMV infection which suggests that memory inflation is driven by proliferation of T_CM_ cells in the lymph nodes (23). This hypothesis was subsequently tested by blocking the egress of T cells from lymph nodes which did not abrogate memory inflation (24). Instead, this study supported a second scenario in which latently infected endothelial cells of the vasculature reactivate circulating MCMV-specific CD8^+^ T cells and thereby maintain inflationary T cell levels (24). Both scenarios assume that latently infected non-haematopoietic cells are the main drivers of CD8^+^ T cell inflation maintenance as these cells are observed to contain the majority of latent viral genomes and selective abrogation of antigen presentation in non-haematopoetic cells completely abolished memory inflation (23, 25). Besides the possibility of continuous viral antigen presentation with infectious viral particles below the detection limit (11, 26), sporadic viral reactivation initiated by local inflammation or decreased immune surveillance (27) is assumed to contribute to the maintenance of the CD8^+^ T cell level (Figure 1). However, how fluctuating antigenic stimuli and individual within-host heterogeneity affect the size and long-term stability of the inflationary T cell pool has not been determined yet.

In this study, we combined mathematical modeling and experimental data to investigate CMV-specific CD8^+^ T cell dynamics within individual mice in more detail. Measuring the dynamics of inflationary and non-inflationary CMV-specific CD8^+^ T cells in the blood of mice over long periods of time, we tested the ability of mathematical models that differ in their assumptions of the viral stimuli regulating T cell expansion for explaining the generation and maintenance of inflationary CD8^+^ T cells. By following the dynamics of the actual cell numbers rather than the frequencies, our analysis allowed a detailed quantification of the different phases of memory inflation, revealing larger heterogeneity in the magnitude rather than the timing of the response phases between individual mice. Furthermore, using our mathematical model for explaining the inflationary CD8^+^ T cell dynamics, we tested the effect of inherent changes in viral reactivation patterns and immune efficacy on the possibility to observe memory inflation. Our analysis provides a first step toward a systematic and mechanistic description of memory inflation dynamics and indicates additional information that might help to elucidate these dynamics.

## Materials and Methods

### Experimental Protocol

Naïve C57BL/6 mice were infected by intravenous injection of 5 × 10^6^ pfu MCMV-Δ157 and blood was repeatedly sampled from the tail vein of individual mice at different time points between day 7 and 70 post infection (p.i.). Cells were analyzed by flow cytometry with respect to their surface marker expression, including markers for CD8, CD44, CD127, CD62L, and KLRG1 and their specificity for the MCMV epitopes M38 (inflationary) and M45 (non-inflationary). Cell numbers per *ml* blood were determined based on extrapolation with a given number of added fluorescently-labeled PE+ beads. Measurements having a living leukocyte percentage lower than 90% or a measured PE+ bead number higher than 10^4^ were excluded from the mathematical analysis, as these values indicated unreliable measurements.

### Ethics Statement

This study was conducted in accordance to the guidelines of the animal experimentation law (SR 455.163; TVV) of the Swiss Federal Government. The protocol was approved by Cantonal Veterinary Office of the canton Zurich, Switzerland (Permit number 127/2011, 146/2014, 114/2017).

### Mathematical Models Describing T Cell Dynamics

We developed different types of models and tested their ability in describing the experimentally observed dynamics of inflationary and non-inflationary T cells. The models differed in the viral stimuli assumed for T cell activation and maintenance in accordance with previous hypotheses on inflationary and non-inflationary T cell dynamics (23, 24).

#### Single Viral Compartment Model (SV)

In the most simple model, we assume that during the time course of the infection virus, *V*, replicates at a constant replication rate ρ_*V*_, and is cleared dependent on the concentration of epitope-specific CD8^+^ T cells, *E*, at rate *kE*. CD8^+^ T cells are activated dependent on the viral load to proliferate with rate ρ_*E*_*V*, and are assumed to be lost from the system at rate δ. The system of ordinary differential equations describing these dynamics is then given by:

(1) $$\begin{array}{l} \frac{dV}{dt}=\rho_{\text{V}}V-kVE\\ \frac{dE}{dt}=(\rho_{\text{E}}V-\delta )E\;. \end{array}$$

Please note, that here the viral replication rate ρ_*V*_ represents a net-replication rate combining viral replication and unspecific clearance. Including the initial value for the CD8^+^ T cells at day 7 p.i. (*E*_7_), the VD-model has five unknown parameters.

#### Latent Viral Reservoir Model (LR)

Accounting for the hypothesis that restimulation of inflationary T cells is mainly triggered by antigen presentation on non-haematopoietic cells (23, 24), we extended the previous SV-model by distinguishing between two different viral compartments. These compartments represent the viral stimuli mediated during acute infection, *V*, and the latent viral reservoir, *R*, related to non-haematopoietic cells that are responsible for antigen-dependent re-activation of CD8^+^ T cells during persistent infection. The latent viral reservoir is assumed to build up during the acute infection phase until reaching a constant level. To account for viral latency, we additionally assume that CD8^+^ T cells are not able to deplete the reservoir. The corresponding system of ordinary differential equations is then given by

(2) $$\begin{array}{l} \frac{dV}{dt}=\rho_{\text{V}}V-kVE\\ \frac{dR}{dt}=\left(\beta V+\rho_{\text{R}}R\right)\left(1-R\right)\\ \frac{dE}{dt}=(\rho_{\text{E}}\left(V+R\right)-\delta )E \end{array}$$

where *V* denotes the viral load during acute infection, and *R* the latent, non-haematopoietic cell related (23, 24) viral reservoir. The net-replication rates of the acute and latent viral reservoir are denoted by ρ_V_ and ρ_R_, respectively. In addition, virus during acute infection is assumed to infect non-haematopoietic cells at rate β. As no data about the viral load is available, the maximal level of the latent reservoir was arbitrarily set to one. CD8^+^ T cells, *E*, are assumed to proliferate proportionally to the overall viral load, *V*+*R*. Together with the initial conditions *V*_7_ and *E*_7_ (with *R*_0_ = 0), the LR-model has eight unknown parameters.

#### Extended Latent Reservoir Model (ELR)

As an additional extension to the *latent reservoir*-model, we assumed that the initial infection of non-haematopoietic cells during acute infection, i.e., the initial generation of the latent reservoir, is not affected by the general carrying capacity of the latent reservoir. Therefore, CD8^+^ T cells can initially receive a stronger stimulus before the non-haematopoietic steady state is reached. The *extended latent reservoir*- model is then defined by:

(3) $$\begin{array}{l} \frac{dV}{dt}=\rho_{\text{V}}V-kVE\\ \frac{dR}{dt}=\beta V+\rho_{\text{R}}R\left(1-R\right)\\ \frac{dE}{dt}=(\rho_{\text{E}}\left(V+R\right)-\delta )E\;. \end{array}$$

As for the LR-model, the ELR-model has eight unknown parameters.

#### Constant Influx Model (CI)

As non-inflationary CD8^+^ T cells are generally observed to decline in numbers after reaching a peak around day 7–8 p.i. (28, 29), we additionally considered a simplified model that accounted for a constant influx Λ of cells into the circulating T cell pool, which neglected the consideration of additional viral activation compartments. This simplifies the model in terms of unknown parameters that need to be considered and leads to

(4) $$\begin{array}{l} \begin{array}{cc} \frac{dE}{dt} & =\Lambda -\delta E\;, \end{array} \end{array}$$

where δ defines the loss rate of circulating CD8^+^ T cells. This standard CI-model has three unknown parameters including the unknown initial concentration of T cells, *E*_0_. Additional extensions to this constant influx model allowing variations for the influx rate Λ are shown in the Appendix A1 and Table S1.

### Parameter Estimation

All models were fitted to the experimental data based on a nonlinear mixed effects model (NLMEM) approach (30). Estimation was performed within Monolix (Version 2016R1, see http://lixoft.com/products/monolix/) using the stochastic approximation expectation-maximization algorithm (31) and assuming a proportional error in the measurements. As viral load cannot be measured, we arbitrarily set the initial value for the viral load for each particular antigen (M38, M45) at day 7 to *V*_7_ = 1/ml with the exception of the LR- and ELR-model. For these models, the viral load was determined relative to the steady-state level of the combined viral load (*V* + *R*), estimating *V*_7_ and defining *R*_7_ = 0.

### Modeling Sporadic Viral Reactivation

To model viral reactivation as a sporadic event in comparison to the continuous stimuli assumed in the mathematical models above, we used a system of impulsive differential equations. Here, the increase of the viral load is modeled in pulses at specified time points *t*_*i*_, *i* ∈ ℕ and is included in the dynamics by adding a specified amount of reactivated virus *R*_*i*_ at each time point *t*_*i*_ to the current amount of virus *V*(*t*_*i*_). The equations are given as

(5) $$\begin{array}{l} \frac{dV}{dt}=-kVE,\;t\neq t_{i}\\ \Delta V=V+R_{i},\;t=t_{i}\\ \frac{dE}{dt}=(\rho_{\text{E}}V-\delta )E\;, \end{array}$$

with the nomenclature of parameters being identical to the SV-model (see Equation 1).

## Results

### Individual Dynamics of CD8^+^ T Cell Responses Against Inflationary and Non-inflationary Epitopes

**Figure 2:**
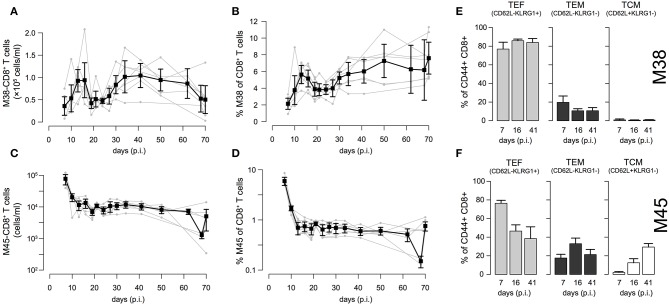
Dynamics of inflationary and non-inflationary CD8^+^ T cells during MCMV infection: **(A,B)** Longitudinal data of the number and frequency of inflationary M38-specific CD8^+^ T cells in the blood for seven different mice. Measurements were obtained for up to 70 days after infection with MCMV. Gray dots and lines indicate the dynamics within the individual mice (*n* = 7) and black squares the mean with corresponding error bars (±1.96×SE) for each time point. **(C,D)** Corresponding measurements for the non-inflationary M45-specific CD8^+^ T cells within the same mice. **(E,F)** Frequency of effector (T_EF_, CD62L^−^KLRG1^+^), effector-memory (T_EM_, CD62L^−^KLRG1^−^) and central memory T cells (T_CM_, CD62L^+^KLRG1^−^) among M38- **(E)** and M45-specific **(F)** activated CD8^+^ T cells for 3 specified time points representing the acute, contraction and long-term memory phase of the responses. For a continuous dynamics of the individual cellular subsets see Figure S1.

To examine the individual dynamics of inflationary and non-inflationary CD8^+^ T cells in the blood, we infected C57L/B6 mice with MCMV-Δ and took repeated blood samples in a time period from 7 to 70 days p.i. that were analyzed for the number of M38- (inflationary) and M45- (non-inflationary) specific CD8^+^ T cells. Despite the heterogeneity between individual mice with respect to actual cell numbers, we generally observed 4 different phases in the dynamics of the inflationary M38-specific CD8^+^ T cell response (Figure 2A): Cell numbers increased and reached a peak around day 13–16 post infection, before declining to roughly 50% of the peak value around day 20 post infection. After a second increase, reaching values similar to the previous peak that are maintained over 15–20 days, the cell numbers were slowly declining again. Corresponding measurements of cell frequencies followed a similar phase-dependent dynamics (Figure 2B), with the last decay in inflationary cell numbers not being reflected within these measurements. In contrast to the inflationary M38-specific T cell response, the non-inflationary M45-specific CD8^+^ T cell response declined after the peak around day 7 p.i. to roughly 10% of the peak value around day 20 post infection. This level was maintained throughout the remaining observation period up to day 70 p.i. (Figure 2C). Here, cell numbers and frequencies showed similar dynamics (Figures 2C,D). The inflationary CD8^+^ T cell response was dominated throughout the infection by cells showing an effector (T_EF_, CD62L^−^KLRG1^+^) and effector-memory (T_EM_, CD62L^−^KLRG1^−^) phenotype, which comprised ~ 80% and 10–15% of the activated inflationary CD8^+^ T cell population, respectively (Figure 2E and Figure S1). In contrast, M45-specific non-inflationary CD8^+^ T cells showed the conventional cellular subset dynamics with effector cells dominating the acute phase, while central memory T cells (T_CM_, CD62L^+^KLRG1^−^) slowly increased in frequency and dominated the long-term response (Figure 2F and Figure S1).

### Determining the Dynamics of Inflationary M38-Specific CD8^+^ T Cells

**Figure 3:**
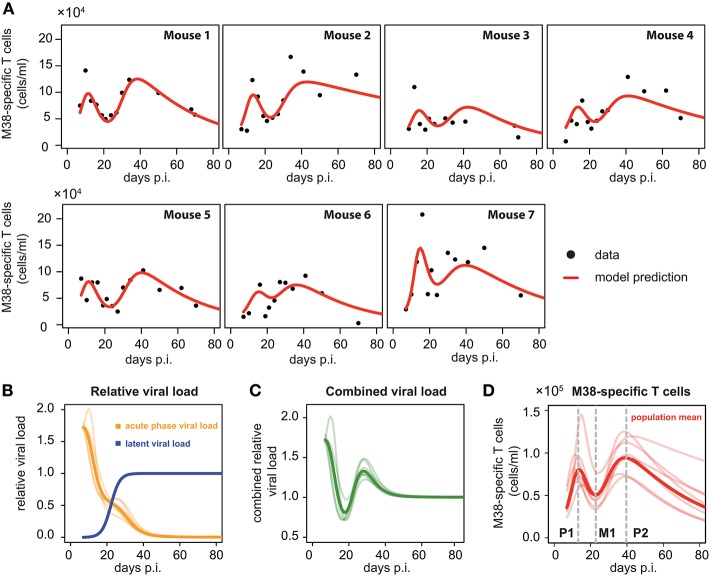
Individual fits to the inflationary M38-specific CD8^+^ T cell response: **(A)** Panels show the model prediction (red) compared to the individual experimental data (black). Model predictions are based on a non-linear mixed effect modeling approach using the ELR-model (see *Materials and Methods* for a detailed explanation of the model). Individual and population-based parameter estimates are shown in Table 2. **(B)** Predicted relative viral load for M38-specific antigen over time distinguishing between an acute, depletable viral load (orange), and the latent viral reservoir (blue). Dynamics is given relative to the level of the latent reservoir at steady state. **(C)** Dynamics of the combined viral load of depletable and latent virus. **(D)** The population and individual dynamics of the M38-specific T cell response indicating the timing of the different phases. Solid lines indicate the population dynamics, opaque lines the individual fits from **(A)**. Vertical gray dashed lines indicate the timing of the peak of the first (P1) and secondary (P2) expansion phase, and the intermediate nadir (M1) indicating the start of the secondary expansion phase.

To compare and quantify the dynamics of the individual CD8^+^ T cell responses in the blood, we tested the ability of different mathematical models in describing the observed dynamics. These mathematical models differed in the viral stimuli assumed to affect the dynamics of the CD8^+^ T cells in the blood. In particular, we distinguished between mathematical models that assumed either a single viral population or two separate viral populations, i.e., acute and latent viral reservoirs, for the activation and re-activation of the CD8^+^ T cell responses. These mathematical models were then fitted to the number of M38-specific CD8^+^ T cells using a non-linear mixed effect modeling approach that accounts for population-based behavior and individual dynamics (see *Materials and Methods*) and their performance in explaining the experimental data was assessed. We found that mathematical models assuming reactivation of the inflationary CD8^+^ T cell population being dependent on a latent-viral reservoir provide better descriptions of the overall cellular dynamics than those assuming a single viral compartment or a constant influx of T cells (Table 1; Figure 3A). The best model for explaining the observed dynamics of the inflationary CD8^+^ T cell response, i.e., the *extended latent reservoir* model (ELR-model), assumes that the latent-viral reservoir specific for M38-activation is limited in size, but that establishment of the reservoir during the acute infection phase is not affected by this size limitation (Equation (3) and *Materials and Methods*). This ELR-model is also preferred to a model that assumes the initiation of the latent viral reservoir being limited by the capacity of the reservoir, which performs slightly worse (ΔAICc = 7).

**Table 1:** Performance of different mathematical models in describing individual CD8^+^ T cell dynamics.

	**Mathematical models describing T cell dynamics**			
**ΔAICc**	**Single-viral compartment (SV)**	**Latent viral reservoir (LR)**	**Extended latent reservoir (ELR)**	**Constant influx (CI)**
M38	15	7	0	14
M45	127	25	43	0

*The table shows the performance of the different mathematical models that were fitted to the number of inflationary (M38) or non-inflationary (M45) CD8^+^ T cells using a non-linear mixed effect modeling approach. Models differed in the assumption of the viral stimuli responsible for T cell activation and re-activation assuming either a single viral population (SV-), or two separated viral populations representing acute and latent viral reservoirs (LR- and ELR-model). In addition, a further simplified model assuming a constant influx of T cells after viral infection (CI-model) was tested. For a detailed description of the individual models see Materials and Methods. Model performance was assessed by the corrected Akaike information criterion (32, 33) and values in the table represent the ΔAICc, i.e., the difference to the AICc-value of the best performing model (ΔAICc=0)*.

The ELR-model captures the secondary expansion dynamics within individual mice and predicts a long-term decline in M38-specific CD8^+^ T cell numbers (Figure 3A). Non-latent M38-specific antigen is estimated to be present up to ~40 days p.i. (Figure 3B). This is in line with findings by Torti et al. (23), in which MCMV was still detectable in the liver and the salivary gland 28 days post infection. The model predicts a change in the clearance of the non-latent viral compartment around 20 days p.i. leading to a reduced clearance rate, which coincides with the establishment of the latent viral reservoir. This latent reservoir is estimated to start increasing around day 15 p.i. and reaches its steady state approximately ten days later (Figure 3B). With these dynamics, the combined viral load of latent and non-latent reservoir resembles a damped oscillation (Figure 3C).

Based on our parameter estimates (Table 2), the model predicts an overall half-life of inflationary M38-specific CD8^+^ T cells in the blood circulation of around 2 days, which mostly reflects the short half-life of the cells during the acute infection and contraction phase. Given the continuous restimulation of cells, the inflationary T cell pool itself is estimated to have a total half-life of 33.7 ±8.2 days (mean ± SE). Individual mice vary especially in the estimated viral expansion dynamics, explaining the different peak levels of the inflationary T cell responses during the first and secondary expansion phases of individual mice (Table 2 and Figure 3D).

**Table 2:** Parameter estimates of the ELR-model for the inflationary M38-specific CD8^+^ T cell response.

	Acute viral load at day 7	T cell concentration at day 7	Net-replication rate of active virus	T cell mediated viral clearance rate	Infection rate of non-haem. cells	Net-replication rate of latent virus	Activation rate of T cells	Death rate of T cells
Mouse	V_7_	E_7_	ρ_*V*_	k	β	ρ_*R*_	ρ_*E*_	δ
	(a.u. ml^−1^)	(× 10^4^cells/ml)	(day^−1^)	(× 10^−6^day^−1^)	(× 10^−4^day^−1^)	(day^−1^)	(day^−1^)	(day^−1^)
1	1.73	6.16	0.15	2.95	5.39	0.414	0.295	0.323
2	1.70	3.94	0.13	3.01	4.79	0.413	0.310	0.318
3	1.79	3.58	0.09	3.12	5.16	0.414	0.289	0.326
4	1.69	3.35	0.10	3.06	4.68	0.413	0.304	0.320
5	1.73	5.58	0.11	3.04	5.15	0.413	0.290	0.324
6	1.70	2.42	0.09	3.14	5.16	0.415	0.290	0.325
7	1.71	3.08	0.18	2.86	5.10	0.414	0.298	0.322
Pop. estimate	1.72	3.60	0.11	3.00	4.90	0.414	0.296	0.322
s.e.	0.06	0.66	0.036	0.55	2.20	0.035	0.03	0.03
relative s.e. (%)	3	18	32	18	45	8	10	10

*Estimates for individual parameters obtained when fitting the ELR-model to the M38-specific CD8^+^ T cell responses using a non-linear mixed effect modeling approach. Estimates for individual mice and the population based parameter estimates (Pop. estimate) including (relative) standard errors (s.e.) are shown. For a detailed description of the model system see Equation (3). (a.u. – arbitrary units)*.

In summary, our analyses support the existence of different viral stimuli early and later during infection to explain the various phases and secondary expansion of the inflationary CD8^+^ T cell response. This supports the hypothesis that the inflationary CD8^+^ T cell response is dependent on viral reactivation and expression of viral genes in latently infected non-haematopoetic cells within circulation or secondary lymphoid organs (23, 24).

### Inflationary CD8^+^ T Cells Show Consistent Timing of the Dynamics in Individual Mice

As has been observed previously (23), the dynamics of inflationary M38-specific CD8^+^ T cells follows a tri-phasic dynamics (Figure 3D): After infection, cells follow a first expansion phase until reaching a first peak that is followed by a contraction phase. After reaching a minimum that is comparable to the initial cell number measured at day 7 p.i., cell numbers increase again until reaching a second peak. Despite variations in the magnitude of the response of individual mice, the timing of these different phases is quite consistent. Based on the model predictions for each individual mouse, the first peak of the inflationary M38-specific CD8^+^ T cell response characterizing the end of the first expansion phase is estimated to occur around 13.21±0.61 days p.i. (mean ± SE) reaching 8.7 × 10^4^ (6.2–14.5 × 10^4^) cells/ml (mean, with numbers in brackets indicating the absolute range of values). The following contraction phase lasts for approximately 12 days with cell concentrations reaching a minimum, which is roughly 1/2 of the cell numbers at the first peak, around day 22.84 (±0.25) post infection. The duration of the secondary expansion phase varies more substantially between mice, with cell numbers reaching a second peak on average 39.66 (±0.83) days post infection. The increase in cells during the secondary expansion phase, i.e., difference between the secondary peak (P2) and the number of cells at the intermediate minimum (M1), correlates strongly with the value at the first peak (P1, *R*^2^ = 0.91, *p*-value = 0.003), excluding mouse 7, for which we observe a massive expansion during the first expansion phase (Figure 3A). However, while the magnitude of the response varies between individual mice, the initiation of the secondary phase that leads to the maintenance of the inflationary responses seems to be quite consistent with respect to its timing.

## Analyzing the Dynamics of Non-inflationary M45-Specific CD8^+^ T Cells

**Figure 4:**
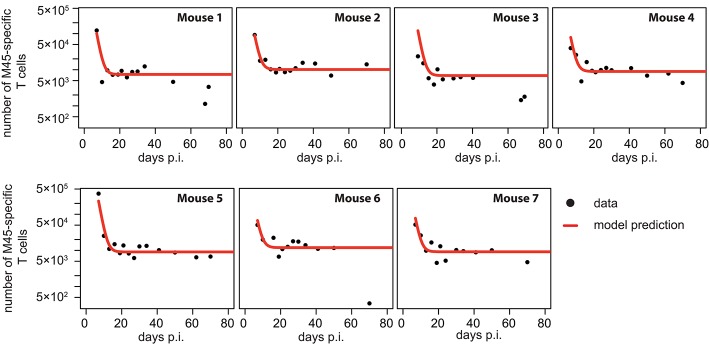
Individual fits to the non-inflationary M45-specific T cell response: Panels show the model prediction (red) compared to the individual experimental data (black). Model predictions are based on a non-linear mixed effect modeling approach using the CI-model (see *Materials and Methods* for a detailed explanation of the model). Individual and population-based parameter estimates are shown in Table 3.

Performing a similar analysis as done for the inflationary CD8^+^ T cell response, we find that the dynamics of non-inflationary M45-specific CD8^+^ T cells is best described by a mathematical model that assumes a constant influx to and loss of T cells from the blood circulation, without the necessity of additional viral stimuli after the acute infection phase (Table 1 and Figure 4). This CI-model provides a sufficient description of the observed dynamics while all other models are not supported, mainly due to the increased number of unknown parameters that cannot be determined based on the available data. In particular, all models explicitly accounting for viral dependent reactivation mechanisms perform worse, indicating a different long-term maintenance of the non-inflationary compared to the inflationary T cell pool.

Based on our parameter estimates, the CI-model predicts an overall half-life for M45-specific CD8^+^ T cells in the blood circulation of around one day (see Table 3), which reflects the death-rate of activated cells during the contraction phase after the resolution of the acute infection (34). Analysis of the CD8^+^ T cell subset distribution shows that non-inflationary T cells are dominated by central and effector memory cells (Figure 2F; Figure S1) which are characterized by a longer half-life (20, 21). Therefore, the estimated influx Λ represents mostly a technical compensation of the model for the estimated loss rate, that is determined by the contraction phase, in order to maintain the observed stable level of cells by homeostatic division during the memory phase. However, recirculation of cells might still play a role (35), but the data do not allow for a separate determination of cell division/influx and death rates during the memory phase.

**Table 3:** Parameter estimates of the CI-model for the non-inflationary M45-specific CD8^+^ T cell response.

Mouse	T cell concentration at day 7	Constant influx of cells	Death rate of T cells
	E_7_	Λ	δ
	(× 10^4^cells/ml)	(× 10^3^cells/ml)	(day^−1^)
1	10.06	4.78	0.646
2	8.87	6.18	0.610
3	11.99	4.33	0.629
4	7.84	5.51	0.616
5	23.21	5.75	0.634
6	6.73	7.11	0.597
7	7.75	5.61	0.613
Pop. estimate	9.48	5.49	0.620
s.e.	2.41	1.00	0.094
relative s.e. (%)	25	18	15

*Estimates for individual parameters obtained when fitting the CI-model to the M45-specific CD8^+^ T cell responses using a non-linear mixed effect modeling approach. Estimates for individual mice and the population based parameter estimates (Pop. estimate) including (relative) standard errors (s.e.) are shown. For a detailed description of the model system see Equation (4)*.

## Various Changes in Sporadic Reactivation Patterns Can Explain Memory Inflation

All mathematical models used so far assumed a constant reactivation to explain the dynamics of the inflationary CD8^+^ T cell response. Due to the maintenance of a viral reservoir, CD8^+^ T cells are continuously reactivated and, thereby, maintain the inflationary T cell pool. However, as many other latent viruses, MCMV is assumed to only reactivate sporadically. If reactivation events are frequent enough, less dense sampling at later time points might prevent the detection of these recurrent reactivations in the data, and could suggest a constant maintenance of the inflationary T cell pool. In case of sporadic reactivations, the dynamics described previously will only represent the mean viral and cellular turnover for the inflationary response.

To assess how sporadic viral reactivations would influence the overall CD8^+^ T cell dynamics, we modified our ELR-model, which was identified to explain the M38-specific inflationary CD8^+^ T cell dynamics. Replacing the previously assumed constant viral reservoir, we used impulsive differential equations (36) to account for various pulses of reactivated latent virus (see *Materials and Methods* for a detailed description of the model). This modeling framework allowed us to test how changes in the reactivation patterns, such as frequency and magnitude of reactivation events, would shape the corresponding CD8^+^ T cell response.

**Figure 5:**
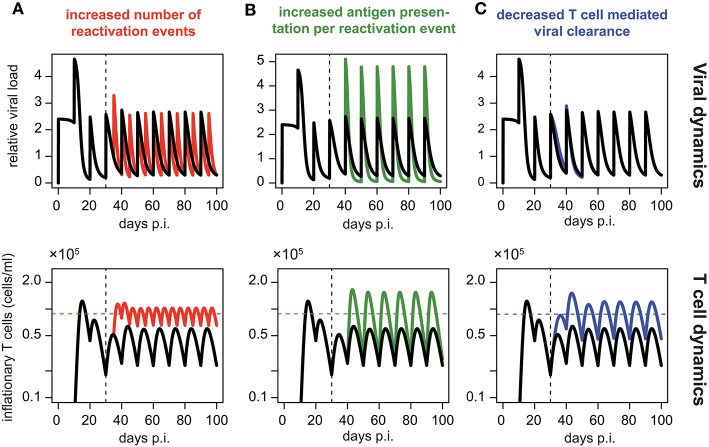
Memory inflation-like dynamics can be caused by changes in viral reactivation patterns or T cell-mediated clearance: Simulated dynamics of viral load (top row) and corresponding T cell dynamics (bottom row) assuming changes in the frequency **(A)** and magnitude **(B)** of viral reactivation events, as well as changes to T cell mediated viral clearance **(C)**. Black lines show the dynamics of the ELR-model adapted for sporadic viral reactivation [Equation (5)] with periodic reactivation patterns every 10 days (Parameterisation: *k* = 5 × 10^−6^, ρ = 0.29, δ = 0.32, *R*_*i*_ = 2.4 and *t*_*i*_ = 10*i, i* ∈ ℕ_0_). **(A)** Effect of increased frequency of reactivation events with viral reactivation occurring within 5 day intervals (red). **(B)** Effect of an increased antigen presentation per reactivation event with (*R*_*i*_ = 4.8, green) which is twice the default value (*R*_*i*_ = 4.8, black). **(C)** The effect of a decreased T cell mediated viral clearance on the dynamics assuming half the efficacy of the default value (*k* = 2.5 × 10^−6^, blue vs. *k* = 5 × 10^−6^, black). Colored horizontal lines indicate the mean number of CD8^+^ T cells from day 50 to 100. Vertical dotted lines indicate the time at day 30 p.i. at which the viral reactivation dynamics changes.

Assuming periodic viral reactivation with the duration between viral bursts and the viral amount released per burst being constant, we found that such a pattern always resulted in a repeated “expansion-contraction” dynamics (see Figure 5, black line), approximating the response observed for non-inflationary T cells. In particular, it does not evoke the observed secondary expansion that lead to a higher secondary peak for inflationary CD8^+^ T cells compared to the first peak of the response (Figure 2D). However, by allowing for changes in the viral reactivation patterns or the CD8^+^ T cell-mediated clearance of infected cells over time, we could recreate the observed memory inflation dynamics in our model in three separate ways: (1) By increasing the frequency of reactivation events, (2) by increasing the amount of antigens produced per reactivation event or (3) by decreasing the CD8^+^ T cell dependent virus clearance rate, *k* (see Figure 5). Allowing for these changes after a certain time, all of these approaches led to the generation and maintenance of a higher long-term level of CD8^+^ T cells compared to a monophasic reactivation pattern.

In summary, these theoretical analyses support our previous finding that memory inflation requires inherent changes in the MCMV reactivation patterns or viral stimuli compared to the acute infection phase.

## Discussion

Understanding the interactions between CMV infection and the adaptive immune system is essential to determine the factors that influence the generation and maintenance of memory inflation. Studies of MCMV infection in mice relying on artificial knock-out strains of mice and viruses already provided important insights into the dynamics of inflationary CD8^+^ T cell responses in different compartments. However, there are still several open questions with regard to the systematic and quantitative interaction of the various factors and processes that are proposed to be involved within the generation and maintenance of the inflationary CD8^+^ T cell pool [reviewed in Klenerman and Oxenius (22)].

In this study, we used time-resolved experimental measurements in combination with different mathematical models to examine and quantify the dynamics of circulating inflationary CD8^+^ T cells within individual mice. Analyzing the dynamics of the number of M38-specific CD8^+^ T cells over time, we find that mathematical models assuming differing viral stimuli during acute infection and the inflationary phase provide a better description of the observed T cell dynamics than models relying on similar viral stimuli during both phases. In contrast, the dynamics of non-inflationary M45-specific CD8^+^ T cells is best described by a model assuming no additional viral stimuli after the acute infection phase, which corresponds to the different processing of M45- and M38-specific epitopes during the infection that either rely on the activity of the immuno-proteasome (M45) or protein processing by the constitutive proteasome (M38), respectively (37, 38). Thus, our mechanistic analysis supports the hypothesis that generation and maintenance of inflationary CD8^+^ T cells is dependent on a latent viral reservoir, e.g., provided by latently infected non-haematopoietic cells in the vasculature or within secondary lymphoid organs as suggested previously (13, 23, 24). This latent viral reservoir responsible for the maintenance of the inflationary response is build up during the acute phase of the infection (0–15 days p.i) and predicted to reach a stable level around 30 days post infection (Figure 3B). Thereby, our analysis favors a model in which the initial generation of the latent reservoir is not affected by a size limitation of the reservoir. This supports current experimental findings that the absolute size of the *T*_CM_ pool generated during priming rather than size constraints within specific compartments determine individual inflationary responses (39).

Our experimental data also support the importance of central memory T cells (T_CM_) for maintaining the inflationary CD8^+^ T cell pool (22, 23): Although we observe on average a 6-7-fold higher absolute number of M38-specific CD8^+^ T cells compared to non-inflationary M45-specific CD8^+^ T cells within individual mice during the memory phase (>21 days p.i.), the absolute number of M38-specific T_CM_ is on average less than 1/3 of the M45-specific T_CM_ cells in the blood (see Figures S1E,F). Thus, reactivation of inflationary CD8^+^ T cells, leading to proliferation and effector-like differentiation of T_CM_ cells is not only indicated by a reduced frequency, but also a lower absolute number of T_CM_ in M38- compared to M45-specific CD8^+^ T cells.

While our mathematical models assumed continuous reactivation of T cells to avoid overfitting of the experimental data, extended analyses investigating the effect of periodic viral bursts on the ability to maintain the inflationary CD8^+^ T cells revealed similar findings concerning the necessity for different viral stimuli during acute and latent infection: To generate and maintain the inflationary CD8^+^ T cell response either viral reactivation patterns in terms of frequency and burst sizes and/or T cell mediated viral clearance rates have to change in comparison to the acute infection phase. Thus, the antigenic stimulation needed to maintain the inflationary CD8^+^ T cell response is suggested to be different than the antigenic stimuli provided during acute infection, in line with previous findings showing that memory inflation does not seem to require full viral reactivation (26). As inflationary CD8^+^ T cells do not show typical signs of T cell exhaustion (22), impaired viral clearance seems to be an unlikely cause for maintaining the inflationary response. The inability of the CMV-specific CD8^+^ T cell response to clear the virus rather plays a role for the establishment of the latent viral reservoir during the acute infection phase, additionally impaired by immune-evasive strategies of CMV (3, 40).

Our analysis indicates different phases in the generation and maintenance of the inflationary CD8^+^ T cell response with consistent timing of these phases between individual mice. A first expansion phase (0–13 days p.i.) is followed by a substantial contraction phase, with secondary expansion starting around ~ 22 days p.i. and reaching a stable level ~ 40 days post infection. While the time point of the predicted initiation of the secondary expansion phase is very consistent between individual mice, the magnitude of the response and the maintained inflationary CD8^+^ T cell level varies substantially (Figures 3A,D). However, our data indicate a strong correlation between the predicted level of the response at the first peak and the novel increase generated during secondary expansion (*R*^2^ = 0.96, *p*-value = 0.003, excluding mouse 7). This supports previous findings that the efficiency of initial viral replication, here indirectly measured by T cell expansion during primary expansion, and corresponding T cell activation during priming dictates secondary memory inflation, i.e., probably regulated by the size of the latently infected cell pool (41) as well as the absolute size of the *T*_CM_ pool that responds to viral reactivation events (39).

The influence of variable antigen load on the extent of memory inflation during MCMV infection has already been shown previously, with lower viral inocula leading to less memory inflation (41) and re-infections stimulating memory inflation (42). While the individual antigen level might also contribute toward explaining the heterogeneity in the magnitude of the inflationary responses for individual mice, the timing of the different phases is largely consistent and, thus, probably independent of the individual antigenic level. Additional factors, such as neutralizing antibodies, also do not play a relevant role during the early phases of MCMV infection (43) that seem to determine the general dynamics of memory inflation (39, 41). However, by affecting the viral load during latency (44, 45), neutralizing antibodies could still influence the magnitude of inflationary responses at later time points. With onset of memory inflation largely dependent on latent infection of non-haematopoietic cells (23, 24) without requiring full viral reactivation (26), viral expression dynamics on these cell populations could be the most relevant factor determining the timing of memory inflation dynamics. However, more detailed and time-resolved analyses, especially during the acute infection phase, are needed to determine the effect of antigen load, antibody levels or concentrations of specific cytokines on the magnitude and timing of individual inflationary T cell responses.

The timing of the individual phases for the inflationary memory response observed in our study slightly deviates from previous findings that indicated a shorter first expansion phase (0-9 days p.i.), immediately followed by a pronounced inflation for M38-specific CD8^+^ T cells (11–15 days p.i.) (23). These differences could partly be explained by the fact that previous observations were made on cell frequencies rather than cell numbers that might be affected by a massive loss of non-inflationary CD8^+^ T cells following the first peak that could still maintain a high frequency of inflationary CD8^+^ T cells despite loss in absolute cell counts.

Our model predicts an overall half-life for inflationary CD8+ T cells in the circulation of around 2 days during the acute infection phase. This rather short half-life is mainly attributed to the loss of effector like T cells that dominate the inflationary response, i.e., reflecting their short half-life (see Figure 2E and Figure S1), which is in line with previous estimates for the half-life of effector T cells during acute infection phases (28, 29). In contrast, given continuous restimulation, the half-life of the total inflationary T cell pool within the circulation during viral latency is estimated to be around ~33 days, varying between individual mice from 19 to 88 days. This corresponds to previous estimates that calculated population half-lifes for transferred T_EM_ in the circulation from around 45–60 days (26) and up to 10–12 weeks within peripheral tissues (21). As many studies show that the overall inflationary pool is maintained in a rather stable manner over long periods of time (13, 23, 24, 46, 47), this indicates constant refueling of the pool of inflationary cells (39). Most of these studies concentrated on the dynamics of the frequency of inflationary CD8^+^ T cells rather than their absolute numbers. While we also observe stable or even increasing frequencies of M38-specific CD8^+^ T cells (Figure 2B) the absolute number of cells is declining at later time points (Figure 2A). To determine if this cell loss would be only temporary and reflects long-term oscillatory cellular dynamics, as e.g., evoked by an increase in viral reactivation events occurring when CD8^+^ T cell levels fall below a certain threshold (3), would require even longer measurements.

In summary, our analyses support previous hypotheses on the importance of viral reactivation for the maintenance of the inflationary memory responses. Carefully determining the dynamics of cell numbers rather than frequencies within individual mice, our analysis revealed multi-phasic dynamics of the inflationary CD8^+^ T cell responses with consistent timing of the individual response phases but variation in the size of the response between mice. Obtaining detailed measurements on cell numbers during acute MCMV infection, as well as denser and longer sampling during the memory phase, might be used to determine differences in the early expansion dynamics of inflationary and non-inflationary T cells, as well as possible oscillatory dynamics in inflationary cell numbers due to viral reactivation events that are invisible when focusing on cellular frequencies. Frequent and dense measurements during the long-term dynamics of the inflationary CD8^+^ T cell response will allow an improved characterization of viral reactivation patterns, identifying the frequency and size of sporadic viral reactivations and possible feedback dynamics of the immune response. This additional information will also help us to infer more detailed mathematical models that provide an improved understanding of the antigen-specific dynamics regulating CD8^+^ T cell responses during MCMV infection.

Our analysis provides a first step toward a systematic description of the dynamics of inflationary CD8^+^ T cell responses within individual mice. Extending the analysis by following the dynamics of the response within other organs and accounting for additional cell populations (46) will help to determine the impact of these various compartments and their systematic interaction on the maintenance of the inflationary CD8^+^ T cell response. Using such mechanistic frameworks to elucidate the interplay of infection dose and individual host factors on the dynamics of memory inflation will provide an important prerequisite for an efficient exploitation of CMV as a vaccine vector against various diseases.

## Data Availability

All relevant data are within the manuscript and raw data are made available upon request.

## Ethics Statement

This study was conducted in accordance to the guidelines of the animal experimentation law (SR 455.163; TVV) of the Swiss Federal Government. The protocol was approved by Cantonal Veterinary Office of the canton Zurich, Switzerland (Permit number 127/2011, 146/2014, 114/2017).

## Author Contributions

AO and FG conceived and designed the study. NB performed the experiments. MG and FG developed the mathematical models. MG, NB, AO, and FG analyzed the experimental data. MG, AO, and FG wrote the manuscript.

### Conflict of Interest Statement

The authors declare that the research was conducted in the absence of any commercial or financial relationships that could be construed as a potential conflict of interest.
