# Supplementary material for: Investigating the Dynamics of MCMV-Specific CD8+ T Cell Responses in Individual Hosts
Source: Front Immunol. 2019 Jun 19;10:1358. doi: 10.3389/fimmu.2019.01358 (PMC6595046; doi:10.3389/fimmu.2019.01358)
Supplement: Supplementary file 1 [file Data_Sheet_1.PDF]

Supplementary Material:
Investigating the dynamics of MCMV-specific CD8$^+$ T cell responses in individual hosts

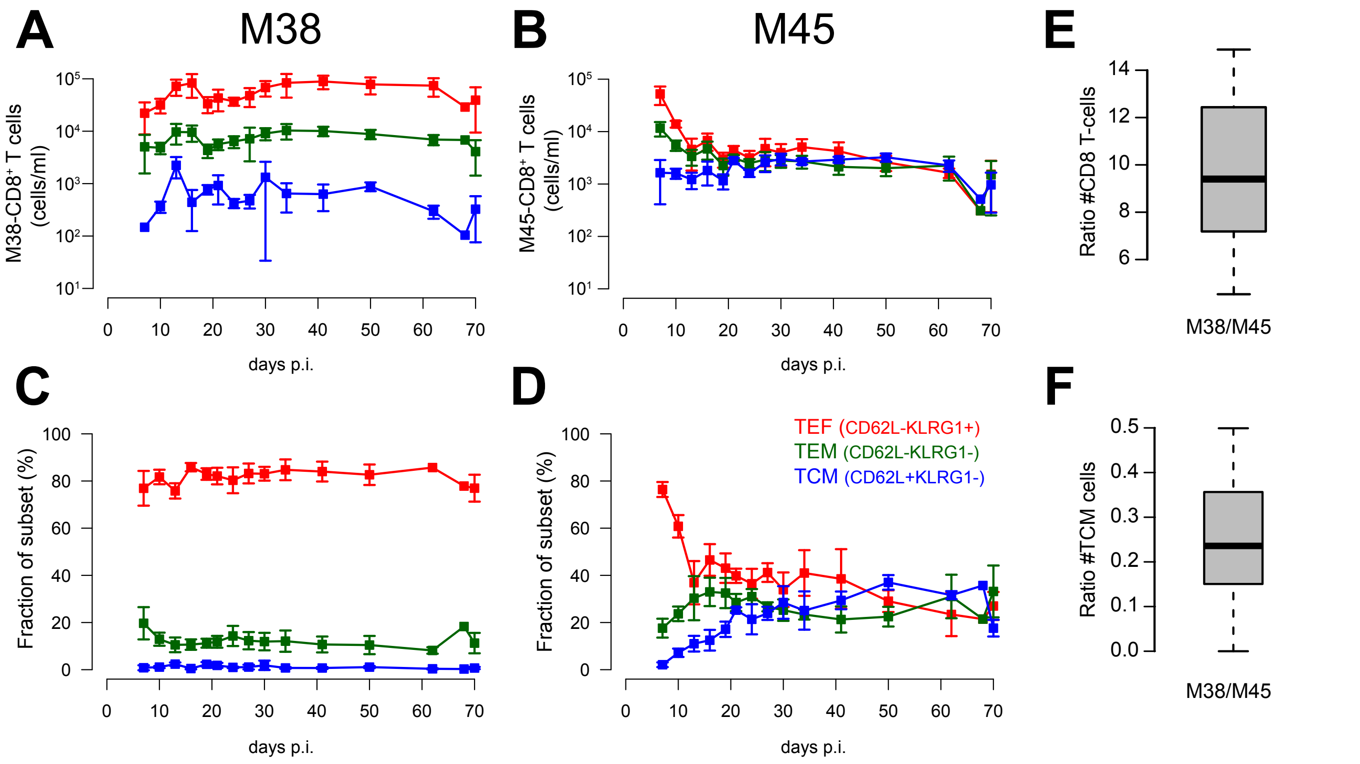

Figure S1. Subset dynamics of MCMV-specific CD8$^+$ T cells: M38- and M45-specific CD8$^+$ T cells were gated for the expression of the surface markers CD62L and KLRG1 to distinguish between effector (TEF, CD62L-KLRG1+, red), effector-memory (TEM, CD62L-KLRG1-, green) and central-memory (TCM, CD62L+KLRG1-, blue) T cells. Individual panels show the dynamics of the number (**A,B**) and fraction of the specific cellular subset among CD44$^+$CD8$^+$ T cells (**C,D**) for inflationary (M38) and non-inflationary (M45) responses, respectively. The mean dynamics showing the mean (filled squares) and deviation ($\pm 1.96 \times$SE, arrows) over all mice is shown. Considering all individual measurements in the long-term memory phase ($\geq$ 30 days p.i), the distribution for the ratio of the absolute number of M38- vs. M45-specific CD8$^+$ T cells of the total response (**E**) and the central-memory T cells (**F**) indicates a significantly reduced number of M38-specific central memory T cells compared to M45-specific T cells despite a substantial larger number of CD8$^+$ T cells.

APPENDIX A1: ADDITIONAL MATHEMATICAL MODELS

In addition to the mathematical model assuming a constant influx of cells throughout the infection (CI-model), we also considered two additional extensions of this model allowing for increased complexity. Instead of actively considering antigenic stimuli regulating T cell expansion, these models use constant influx rates to account for T cell expansion, i.e., representing additional influx of T cells that have been reactivated elsewhere.

Biphasic influx model (BI):

Instead of assuming a constant influx rate, in a first extension of the CI-model we considered a biphasic influx rate which then changes the system to:

$$\begin{aligned} \Lambda &= \begin{cases} \lambda_1, \ t < T \\ \lambda_2, \ t \geq T \end{cases} \\ \frac{dE}{dt} &= \Lambda - \delta E \ . \end{aligned} \tag{S1}$$

Here, λ_1 and λ_2 define the two different influx rates and T the time point at which the influx rates change. Together with the initial conditions for E_7, the BI-model has five unknown parameters.

Variable influx model (VI):

Additionally expanding the BI-model to allow for more dynamic changes of the influx of cells over time, we developed a model that describes the external supply by a continuous function:

$$\begin{aligned} \frac{d\Lambda}{dt} &= \begin{cases} \alpha(\lambda_1 - \Lambda), \ t < T \\ \alpha(\lambda_2 - \Lambda), \ t \geq T \end{cases} \\ \frac{dE}{dt} &= \Lambda - \delta E \end{aligned} \tag{S2}$$

Here, λ_1 and λ_2 define the maximal influx rates for the two considered influx periods that change at time T, and α a scaling factor that regulates how quickly these maximal influx rates are reached. Together with the initial conditions for Λ and E, the VI-model has seven unknown parameters.

Model performance was assessed by the corrected Akaike Information criterion (AICc) and results are shown in Table S1.

	**Mathematical models**	
Δ AICc	Biphasic influx (BI)	Variable influx (VI)
**M38**	20	7
**M45**	8	16

Table S1. Performance of the extended influx models in describing individual CD8$^+$ T cell dynamics: The table shows the performance of the extended influx models that were fitted to the number of inflationary (M38) or non-inflationary (M45) CD8$^+$ T cells using a non-linear mixed effect modelling approach. Model performance was assessed by the corrected Akaike information criterion (Burnham and Anderson, 2003; Akaike, 1973) and values in the table represent the ΔAICc, i.e. the difference to the AICc-value of the best performing model (ΔAICc=0, compare Table 1).
